# p62 /SQSTM1 coding plasmid prevents age related macular degeneration in a rat model

**DOI:** 10.18632/aging.101537

**Published:** 2018-08-28

**Authors:** Nataliya G. Kolosova, Oyuna S. Kozhevnikova, Darya V. Telegina, Anzhela Zh. Fursova, Natalia A. Stefanova, Natalia A. Muraleva, Franco Venanzi, Michael Y. Sherman, Sergey I. Kolesnikov, Albert A. Sufianov, Vladimir L. Gabai, Alexander M. Shneider

**Affiliations:** 1 Institute of Cytology and Genetics, SB RAS, Novosibirsk, Russia; 2 Novosibirsk State Regional Clinical Hospital, Novosibirsk, Russia; 3 CureLab Oncology, Inc, Deadham, MA 02492, USA; 4 School of Biosciences, University of Camerino, Camerino, Italy; 5 Department of Molecular Biology, Ariel University, Ariel, Israel; 6 Sechenov First Moscow State Medical University, Moscow, Russia; 7 Russian Academy of Sciences, Moscow, Russia; 8 Lomonosov Moscow State University, Moscow, Russia; 9 Research Center of Family Health and Reproduction Problems, Irkutsk, Russia; 10 Department of Biochemistry, Boston University School of Medicine, Boston, MA 02118, USA; 11 Federal Center of Neurosurgery, Tyumen, Russia; * Equal contribution

**Keywords:** p62/SQSTM1, age-related macular degeneration, inflammation, gliosis, OXYS rats, aging, retina

## Abstract

P62/SQSTM1, a multi-domain protein that regulates inflammation, apoptosis, and autophagy, has been linked to age-related pathologies. For example, previously we demonstrated that administration of p62/SQSTM1-encoding plasmid reduced chronic inflammation and alleviated osteoporosis and metabolic syndrome in animal models. Herein, we built upon these findings to investigate effect of the p62-encoding plasmid on an age-related macular degeneration (AMD), a progressive neurodegenerative ocular disease, using spontaneous retinopathy in senescence-accelerated OXYS rats as a model. Overall, the p62DNA decreased the incidence and severity of retinopathy. In retinal pigment epithelium (RPE), p62DNA administration slowed down development of the destructive alterations of RPE cells, including loss of regular hexagonal shape, hypertrophy, and multinucleation. In neuroretina, p62DNA prevented gliosis, retinal thinning, and significantly inhibited microglia/macrophages migration to the outer retina, prohibiting their subretinal accumulation. Taken together, our results suggest that the p62DNA has a strong retinoprotective effect in AMD.

## Introduction

Age-related macular degeneration (AMD) is the most common cause of irreversible vision loss in industrialized countries [1]. Prevalence of AMD is increasing dramatically as the proportion of the elderly in the population continues to rise [1]. By clinical signs, there are two forms of AMD: dry and wet AMD forms, also known as geographic atrophy and exudative AMD, respectively. There are effective treatments of vascular complications of neovascular AMD by anti-VEGF therapeutics. However, neither there is a treatment of the dry form of AMD (~ 90% of all cases) nor preventive strategies against progression to the nonexudative form of AMD [2]. Therefore, the development of effective therapeutic and prophylactic modalities against AMD is an urgent task. AMD is a multifactorial disease involving a complex interplay of genetic, environmental, metabolic, and functional factors [3]. Age-related alterations in the immune system, inflammation and autophagy as well as oxidative stress strongly interwoven into AMD pathogenesis and represent possible targets for new therapies [4].

To study age-associated disorders, we introduced a model of senescence-accelerated OXYS rats, which spontaneously develop a phenotype similar to human AMD-like retinopathy [5–10]. Retinopathy that develops in OXYS rats even at a young age corresponds (in terms of clinical manifestations and morphological characteristics) to the dry atrophic form of AMD in humans. Furthermore, neovascularisation develops in some (~10–20%) of these rats with age. The clinical signs of AMD-like retinopathy appear by the age of 3 months in 100% of OXYS rats against the background of a reduction in the transverse area of the RPE and impairment of choroidal microcirculation [7,8]. Significant pathological changes in the RPE as well as clinical signs of advanced stages of retinopathy are evident in OXYS rats older than 12 months [6,10]. These changes manifest themselves as excessive accumulation of lipofuscin and amyloid in the RPE regions [6], disturbances in the morphology of the RPE sheet, including an increase in the proportion of multinucleated cells, hypertrophy, distortion of cell shape, and reactive gliosis [10]. This animal model is successfully used to study the pathways and molecular alterations implicated in the development and progression of age-related diseases [11,12] as well as to test new therapeutic interventions [8,13,14].

The adapter protein p62/SQSTM1 interacts with many signaling factors, and regulates major cellular functions including inflammation, apoptosis, and autophagy [15,16]. In the retinal pigment epithelium (RPE), p62 promotes autophagy and simultaneously enhances an Nrf2-mediated antioxidant response to protect against acute oxidative stress [16]. Recently, a DNA plasmid encoding p62-SQSTM1 (p62DNA) has been developed as biological agent for treatment diseases associated with chronic inflammation. Indeed, it demonstrated strong anti-osteoporotic activity [17], and alleviated diet-induced obesity and metabolic dysfunctions [18] in animal models. Notably, suppression of osteoporosis and metabolic syndrome by p62DNA were associated with a sharp down-regulation of master pro-inflammatory cytokines, and up-regulation of anti-inflammatory species [17,18]. Because of a significant inflammatory component in AMD, we hypothesized that quenching chronic inflammatory reactions upon administration of p62DNA may alleviate the disease. Here, we assessed effects of p62DNA administration on the development of retinopathy in OXYS rats and evaluated possible mechanisms of its action.

## RESULTS

### p62DNA inhibits retinopathy development in OXYS rats

**Figure 1:**
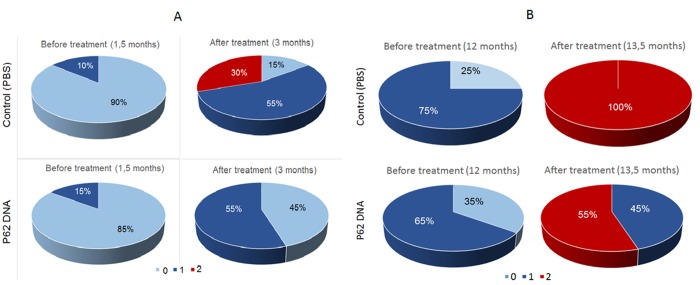
**Effect of treatment with p62 - plasmid on the retinopathy developing in OXYS rats at 1.5 and 12 months of age.** The data are presented as percentage of eyes with stages (0, 1 and 2) of retinopathy before and after treatment in control (PBS) and p62-treated OXYS rats. In each group, 20 eyes of 10 animals were examined.

In the first series of experiments, we assessed possible prophylactic effects of p62DNA against the development of retinopathy. Set of six p62DNA weekly injections started at the age of 1.5 months prior to any signs of retinopathy. Preliminary examination of rats at the age of 1.5 months showed that in experimental and control groups of OXYS rats signs of the 1st stage (1 a.u.) of retinopathy were present in 15 and 10% of animals, respectively. Five injections of the p62DNA ones a week (from 1.5 months of age) significantly slowed down development of retinopathy in OXYS rats (Figure 1A). Indeed, by the age of 3 months, 55% of eyes in the control group developed the signs of the 1st stage of retinopathy, 30% developed the 2^nd^ stage, and only 15% of eyes remained without the signs of the disease. In contrast, in the p62DNA treated group, 55% of eyes developed the signs of the 1st stage of retinopathy, while the rest 45% of eyes did not show any signs of degeneration. Accordingly, statistical analysis showed that the average level of retinopathy in the p62DNA-treated OXYS rat’s eyes was 2.5 times lower than in the control animals (0.45±0.11 and 1.15±0.15 a.u., p < 0.001, respectively).

Another experiment was conducted testing if the plasmid effects progression of AMD in the older animals (Figure 1B). Examination of these animals at the age of 12 months revealed that all animals had signs of retinopathy in at least one eye. 75 percent of eyes in the control group manifested changes corresponding to the AMD predisciform stage (1 a.u.) and 25% did not have the signs of retinopathy. In the experimental group, 65% of eyes had changes corresponding to the predisciform stage (1 a.u.) and 35% of rats did not have the signs of retinopathy. Statistical analysis showed that retinopathy continued to progress in both control and experimental groups but p62DNA reduced the severity of pathological changes in the eyeground of OXYS rats (p < 0.001). By the time of the second eye inspection at the age of 13.5 months, all the eyes in the control group had signs of retinopathy corresponding to the 2nd stage of AMD (2 a.u.). At the same time, p62DNA-treated OXYS rats demonstrated pathological changes corresponding to the 1st stage of AMD in the 45% of retinas, and to the 2st stage - in the 55% of eyes. These data indicate that administration of p62DNA in the prophylactic setting significantly delays development of AMD signs and alleviates the severity of the disease.

### Effect of p62DNA remained for 6 months after the treatment

**Figure 2:**
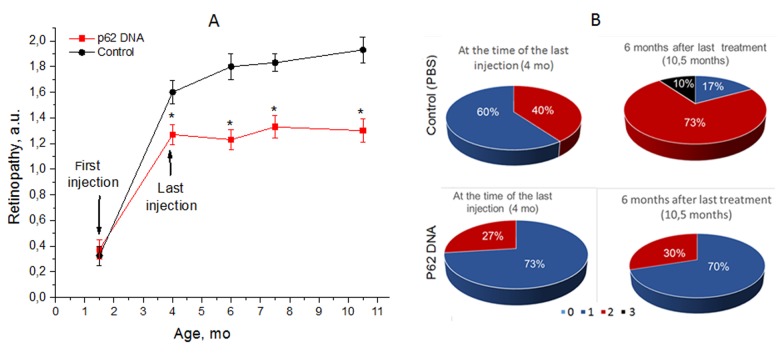
**p62 DNA suppressed development of retinopathy and the effect persisted for 6 months after the last injection.** (**A**) The data are presented as a.u. corresponding to the stages of retinopathy. *A significant increase in the severity of retinopathy according to the pairwise comparisons of the eye condition before and after treatment. (**B**) Stages of retinopathy in 4- and 10.5-month-old controls and p62 treated OXYS rats. Treatment was started at the age 1.5 months. In each group, 30 eyes of 15 animals were examined. The data are presented as the percentage of eyes with stages (0, 1, 2 and 3) of retinopathy.

To assess the duration of the effect of p62DNA on AMD, groups of OXYS rats were administered weekly injections of either p62DNA or PBS control and then observed for 6 months. The first injection took place at the age of 1.5 month, and the last one at 4.5 months. Each animal was examined by an ophthalmologist every second week. The results of examination are shown in Figure 2. The first (preliminary) examination of rats at the age of 1.5 months revealed that the same percentage of eyes in the experimental and the control group of OXYS rats had signs of the first stage of retinopathy (30% and 35% respectively). At the age of 4 months, 73% of eyes manifested signs of 1^st^ stage retinopathy, and 27% - signs of the 2^nd^ stage disease in p62-treated rats. At the same time in control rats, we found signs corresponding to the first and second stages of the disease in 40% and 60% of eyes respectively. According to the ANOVA analysis, an averaged stage of retinopathy in p62-treated rats was significantly reduced compared to the control (p<0.009).

Starting 4 months of age, p62DNA completely prevented further development of retinopathy in OXYS rats. As a result, the severity of retinopathy signs at the age of 10.5 months remained at the level of 4-month-old animals: 70% of eyes of OXYS rats from this group had signs of first-stage and 30% second stage of retinopathy, indicating the disease remained stable during at least 6 months following the p62DNA injections. In contrast, examination of the control animals at the ages of 7.5 and 10.5 months indicated enhancements of the severity of pathological changes (p<0.015). Indeed, at the age of 10.5 months, we detected signs of first-stage AMD in 17% of the eyes, second-stage - in 73%, and the third-stage - in 10% of eyes of the control OXYS rats (Figure 2). Therefore, administration of p62DNA precludes further disease progression, an effect that can last for 6 months.

### Administering of p62DNA does not change expression of retinal p62

**Figure 3:**
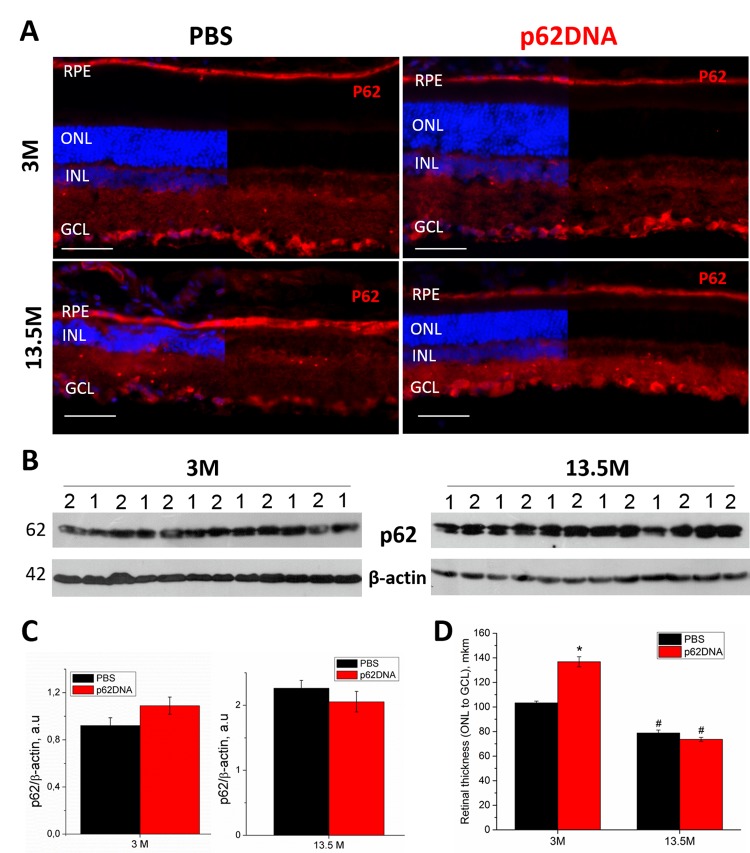
**Effect of p62 DNA on p62 expression in the retina of OXYS rats at 3 and 13.5 months.** (**A**) Representative p62 immunofluorescence of retinal cryosections from 3- and 13.5-month-old OXYS rats treated by PBS (left) or p62DNA plasmid (right). Scale bar: 50 μm. RPE: retinal pigment epithelium; ONL: outer nuclear layer; INL: inner nuclear layer; GCL: ganglion cells layer. (**B**) Representative immunoblots of p62 in the retina of OXYS rats. 1 - PBS; 2 - p62DNA. (**C**) Levels of p62 protein by immunoblot. (**D**) Measurements of retinal thickness (from ONL to GCL) in 3- and 13.5-month-old OXYS rats treated by p62DNA or PBS. *p < 0.05, statistically significant effect of p62DNA; #p < 0.05 between 3 and 13.5 months. Data are presented as mean ± SEM.

Western blot analysis and immunohistochemistry were performed to determine expression of p62 in the retina of 3- and 13.5-month-old OXYS rats receiving injections of PBS (vehicle control) or p62DNA (Figure 3A-C). Immunohistochemical staining of the retinal slices revealed strong p62 expression in the RPE cells and around the nuclei of the inner nuclear (INL) and ganglion cells layers (GCL) in both control and treated animals. P62 expression was weaker in outer and inner plexiform layers (OPL and IPL) in rats of all groups (Figure 3A) (n=4 p62DNA, n=4 PBS). Also, immunostaining revealed p62 granules in plexiform layers, and the number of these granules increased with age. However, we did not detect any significant differences in the p62 immunostaining between plasmid-treated and PBS groups. The lack of difference in expression of p62 protein in rat retina in control and following p62DNA administration either at 3 months or 13.5 months old animals was further confirmed by immunoblotting with anti-p62 antibody (Figure 3B-C) (n=6 p62DNA, n=6 PBS).

### Administering p62DNA prevents degeneration of neuroretina and RPE

We observed a higher overall retinal thickness (from GCL to ONL) in young OXYS rats treated with p62DNA compared to PBS-treated group (Figure 3D) (p<0.05, n=4 p62DNA, n=4 PBS). In the control OXYS rats group, we observed a substantial reduction of the number of rows of photoreceptors (Figure 3A) and the retinal thickness (Figure 3D) by 13.5 months of age. These changes indicate progressive retinal neurodegeneration. At the same time, the age-associated reduction of the number of photoreceptor rows observed at the age of 13.5 months was substantially smaller in animals that received 6 weekly injections of p62 from 12 months. However, treatment of the older rats with the plasmid (starting at the age of 12 months) did not prevent or reverse the decline in retinal thickness.

**Figure 4:**
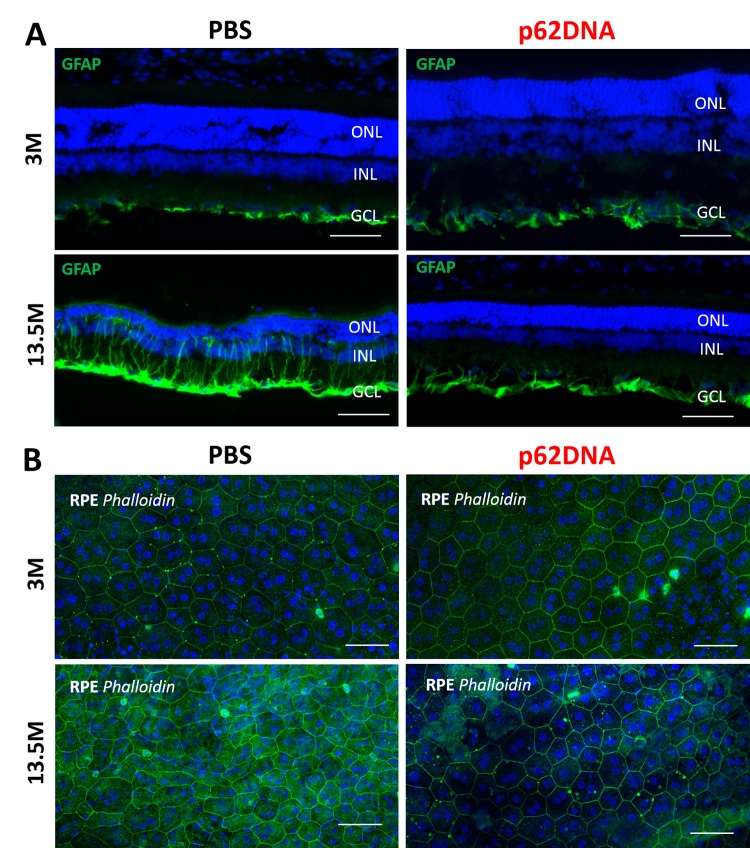
**Effect of p62DNA on the GFAP expression and the state of RPE cells.** (**A**) Representative GFAP immunostaining in retina of 3- and 13.5-month-old OXYS rats, treated by PBS (left) or p62DNA (right). GFAP staining was mainly confined to astrocytes and the ganglion cell layer at the inner limiting membrane in OXYS rats at the age of 3 months. In PBS-treated 13.5-month-old OXYS rats, the increased GFAP expression was observed along the Müller glial cell processes extending towards the outer limiting membrane, representing massive gliosis. p62DNA treatment prevented GFAP accumulation in 13.5-month-old OXYS rats. Scale bar: 50 μm. ONL: outer nuclear layer; INL: inner nuclear layer; GCL: ganglion cells layer. (**B**) Representative images of phalloidin-stained RPE flat-mounts of 3- and 13.5-month-old OXYS rats, treated by PBS (left) or p62DNA (right). p62DNA treatment slowed down development of the destructive alterations of RPE cells (the loss of regular hexagonal shape, the hypertrophy, the multinucleation) in OXYS rats. Scale bar: 50 μm.

RPE cells are first affected during AMD pathogenesis. In line with this observation, destructive alteration in RPE cells is a primary change during the development of retinopathy in OXYS rats [10]. We investigated the effect of p62DNA on the state of actin cytoskeleton in RPE cell by staining RPE flat mounts with phalloidin (Figure 4B). According to the commonly accepted practice assuring the highest quality and reproducibility, we analysed only the central zone of the RPE, which is in close proximity to the exit site of the optic nerve. In 3 months old PBS control OXYS rats, we observed hypertrophic and multinucleate RPE cells with loss of hexagon shape, indicating significant abnormalities. By the age of 13.5-months in PBS control OXYS rats, the majority of RPE cells displayed disorganized morphology and the loss of hexagon shape. This qualitative assessment showed a significant increase in proportion of multinucleate and hypertrophic RPE cells upon aging. In contrast, in p62DNA treated groups, the RPE cell exhibited mostly a regular organized structure with a smaller proportion of pathologically altered cells (Figure 4B). Thus, p62DNA treatment significantly alleviated destructive alterations of RPE cells.

### Effect of administering p62DNA on GFAP expression

Upregulation of glial fibrillary acidic protein (GFAP) is a well-established indicator of retinal injury and reactive gliosis [19]. Accordingly, we investigated expression of GFAP by immunohistochemistry with the corresponding antibody. At the age of 3 months, GFAP staining was mainly confined to astrocytes and ganglion cell layer at the inner limiting membrane (Figure 4A), and there was no significant difference between levels of GFAP in the retina of p62DNA and PBS groups. By the 13.5 months of age, astrocytes and Müller cells were strongly activated in control animals, as shown by the intense GFAP staining in the macroglial outgrowths from GCL towards the outer limiting membrane beyond ONL, representing massive gliosis (Figure 4A). However, p62DNA treatment strongly reduced, and in some cases completely prevented GFAP upregulation in 13.5-month-old OXYS rats (Figure 4A) (n=4 p62DNA, n=4 PBS). Therefore, administration of p62DNA has a strong preventive effect on multiple hallmarks of developing AMD.

### Effect of p62DNA on retinal microglia and macrophages

**Figure 5:**
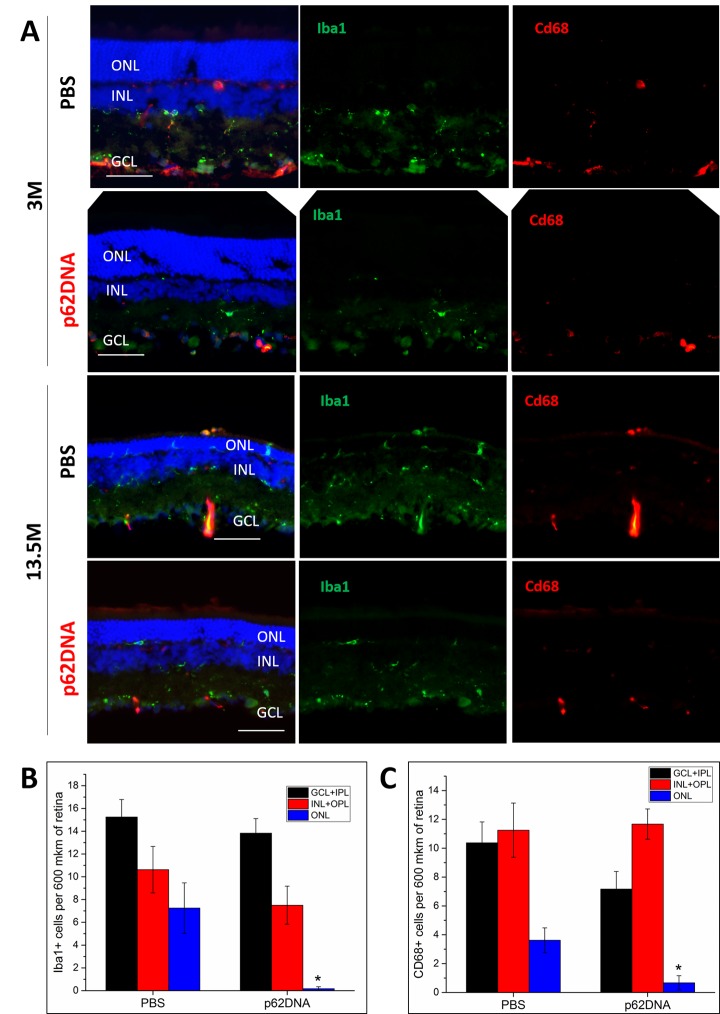
**p62DNA abolished migration of Iba1+ and Cd68+ positive cells (microglia and macrophages) to the outer nuclear layer in 13.5-month-old OXYS rats.** (**A**) Representative images of Iba1 (green) and Cd68 (red) immunostaining in OXYS rats treated with PBS or p62DNA at 3 and 13.5 months of age. The distribution of Iba1^+^ (**B**) and Cd68^+^ cells (**C**) in various layers of the retina: ganglion and inner plexiform layers (GCL+IPL, black), inner nuclear and outer plexiform layers (INL+OPL, red) and outer nuclear layer (ONL, blue). Scale bar: 50μm. ONL: outer nuclear layer; INL: inner nuclear layer; GCL: ganglion cells layer. *p < 0.05, statistically significant effect of p62DNA. Data are presented as mean ± SEM.

In order to evaluate effects of the p62DNA on the inflammatory conditions in the retina and to quantify recruitment of microglia and macrophages to the outer retina, we performed double immunostaining of retinal cryosections with Iba1 (a microglial marker) and Cd68 (a marker of macrophages). Figure 5 depicts representative images of Iba1 and Cd68 immunoreactivity on retinal cryosections. At the age of 3 months, the Iba1^+^ and Cd68^+^ cells were located in the GCL, INL, IPL and OPL. At this age, we did not detect migration of microglia and macrophages into the photoreceptor layer, and p62DNA treatment did not affect the amounts Iba1 and Cd68 cell (Figure 5A-C). By the age of 13.5 months, PBS treated OXYS rats displayed Iba1^+^ and Cd68^+^ cells in ONL, indicating inflammatory processes (Figure 5C). In contrast, treatment with p62DNA almost completely blocked appearance of microglia and macrophages in the ONL, indicating little or no retinal inflammation following the p62 injection (Figure 5C) (p<0.05, n=4 p62DNA, n=4 PBS). Overall these data indicate that p62 administration alleviates development of the age-related chronic inflammation and reverses retinal degeneration in the rat model of AMD.

## DISCUSSION

Herein, we assessed preventive and/or therapeutic potency of a plasmid coding p62-SQSTM1. We report that administering the p62DNA to young OXYS rats substantially blocked AMD-like retinopathy limiting the disease signs. Unlike many other interventions manifesting withdrawal effect, the prophylactic effect of p62 DNA in young animals persisted for half a year after the last injection. The therapeutic effect was tested administering p62DNA to the rats at the age of 12 months when retinopathy has already actively progressed. Indeed, administering p62DNA to these animals suppressed further disease development. Therefore, the plasmid coding p62-SQSTM1 is a promising anti-AMD agent.

Degeneration and loss of RPE and choroidal involution with a secondary loss of photoreceptors are cardinal features of the dry form of AMD. Our prior data indicate that AMD-like pathology in OXYS rats is strongly associated with age-related alterations of the RPE and glia, and may derive from an imbalance of immune processes, including chronic inflammation [10,11]. Normal RPE sheet is organized as a regular array of cells while upon AMD it exhibits strong spatial irregularity [20]. Other RPE changes typical for AMD include hypertrophy, multinucleation, and disruption of the hexagonal structure. Importantly, administering p62DNA prevented development of the destructive alterations of RPE cells (Figure 4) and thinning of the retina. Also, it reduced the loss of photoreceptor neurons, thus providing a neuroprotective effect in OXYS retina (Figure 3). To some extent, neuroprotection observed in AMD OXYS rat model may be due to primary protection of the RPE integrity.

Many diseases of retina are related to the gliosis of Müller cells and astrocytes, and upregulation of GFAP is a well-established indicator of retinal injury and reactive gliosis [21]. In AMD, regions of GFAP upregulation in Muller cells are associated with drusen formation [22]. Importantly, inhibition and/or reversal of the reactive gliosis prevents apoptotic death of retinal neurons and provides substantial neuroprotection [23,24]. Here, we demonstrate that treatment with p62 DNA substantially prevents GFAP activation and decreases reactive gliosis in retina of OXYS rats.

**Figure 6:**
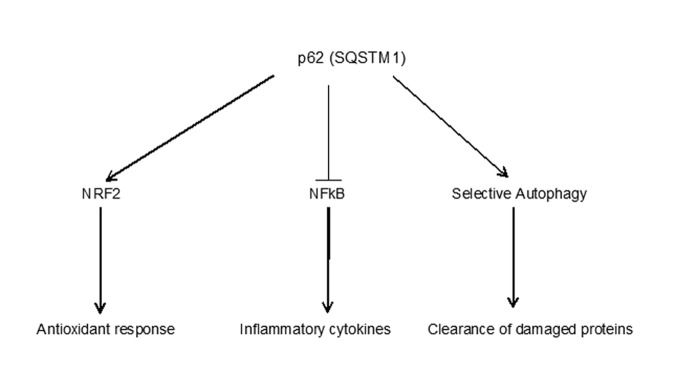
**p62 (SQSTM1) as a mediator of several pathways.** Anti-inflammatory effect of p62 can be mediated via inhibition of NF-kB pathway as well as antioxidant response and clearance of damaged proteins/organelles (e.g., mitochondria) [25–28].

In the present study we demonstrated that in aged OXYS rats macrophagal and microglial infiltration in outer retina (the number of Iba1- and Cd68-positive cells) was reduced in p62DNA treated rats compared to PBS control, which is consistent with the previously proposed anti-inflammatory protective role of p62 plasmid. Recently, we reported that p62-encoding plasmid administered intramuscularly reduces levels of pro-inflammatory cytokines, increases levels of anti-inflammatory cytokines and mitigates inflammation-related diseases such as osteoporosis and metabolic syndrome [17,18]. There are two ways how administering p62DNA can lead to the anti-inflammatory effect, either directly influencing p62 level in the cells of the target organ or acting indirectly. Cells of the mammal organisms naturally express p62, also known as p62/sequestosome-1 (p62/SQSTM1), which plays a variety of biological roles ranging from oxidative stress, tumorigenesis, autophagy and degradation of misfolded proteins to inflammation and anti-inflammatory response (Figure 6) [16,25]. For instance, p62 inhibited MYD88-TRAF6 complex formation, suppressing expression of IL-6 and nitric oxide synthase 2 [26]; and p62 overexpression decreased inflammatory cytokines production [27]. Also, inflammation may be controlled by interplay between the p62 level and NF-kB activity [28] (Figure 6). Interestingly, mice deficient in p62 developed more severe atherosclerosis and showed greater macrophage infiltration of atherosclerotic plaques, a sign of increased inflammation [29]. Thus, if administering p62DNA leads to increased local expression of p62 in the organ, it may reduce local inflammation.

The suppression of osteoporosis by the p62DNA was associated with up-regulation of endogenous p62 protein in bone-marrow stromal cells [17]. However, in the present study, we did not detect elevation of p62 levels in the RPE/retina of the p62DNA treated groups. Therefore, tissue protection and suppression of inflammation can be achieved through a mechanism that does not involve elevation of endogenous p62 levels. Effects of p62DNA in this model seem to be indirect. The likely scenario is that the plasmid enters some cells remotely from the target organ, and expression of the plasmid-encoded p62 in these remote cells generates signals circulating in the body and causing therapeutic effects by reducing local inflammation. Thus, we believe that our work is fundamentally different from a direct gene therapy approach of Caccamo et al. [30] who have shown that increasing brain p62 expression might be a valid approach to restore neuronal function in proteinopathies.

Discovering the anti-inflammatory signal(s) induced by the p62DNA may be an important continuation of this line of research. A signal may be a secreted molecule, an exosome or even a reprogrammed cell. Although majority of the administered plasmid molecules do not leave the muscle they are injected to, some plasmid is delivered to a bone marrow. This fact lead to a hypothesis [18] that the plasmid transfects macrophages in the bone marrow shifting them to the anti-inflammatory M2-like phenotype. Then, these macrophages exit the bone marrow and circulate in the body.

In summary, our data suggests that a p62-encoding plasmid might be a novel preventive and/or therapeutic agent for AMD as it maintained retinal thickness and restored RPE morphology.

## MATERIALS AND METHODS

### DNA Plasmid

Human p62 (SQSTM, isoform 1) – encoding DNA vaccine (Elenagen) was previously described [31] and produced using EndoFree Plasmid Giga Kit (Qiagen).

### Animals and treatments

All experimental procedures were in compliance with the European Communities Council Directive of 24 November 1986 (86/609/EEC). The protocol of the animal study was approved by the Commission on Bioethics of the Institute of Cytology and Genetics, Novosibirsk, Russia. Male senescence-accelerated OXYS rats were obtained from the Center for Genetic Resources of Laboratory Animals at the Institute of Cytology and Genetics, the Siberian Branch of the Russian Academy of Sciences. The OXYS strain was derived from the Wistar strain of rats at the Institute of Cytology and Genetics as described earlier [9]. At the age of 4 weeks, the pups were weaned, housed in groups of five animals per cage (57 × 36 × 20 cm), and kept under standard laboratory conditions (22°C ± 2°C, 60% relative humidity, and 12-hour light/12-hour dark cycle; lights on at 9 a.m.). The animals were provided with standard rodent feed (PK-120-1; Laboratorsnab, Ltd., Moscow, Russia) and water *ad libitum*.

### *The first experiment*

OXYS rats at the age of 1.5 months (n=20) and 12 months (n=20) were distributed in four groups (n = 10) and were injected intramuscularly (femoral quadriceps) with p62DNA in dose 150 mkg per rat in 60 µl (Elenagen, 2.5 mg/ml) on phosphate-buffered saline (PBS) or with only PBS. All groups were subjected to five injections at one-week intervals. Ophthalmoscopic examination was carried twice: before and 2 weeks after the last plasmid injection. The rats were euthanized using CO_2_ inhalation and decapitated 8 d after the last examination of eyes. Eyes from four rats for group were used for immunohistochemistry (the right eyes) and RPE flat-mount staining (the left eyes). At least four tissue slices were analyzed per animal. To measure p62 protein level we used retinas obtained from six rats for group (the left and right eyes were mixed). The retina was separated from the other tissues, placed in microcentrifuge tubes for protein isolation, and frozen in liquid nitrogen. All specimens were stored at −70°C before the analysis.

### *The second experiment*

1.5-month old OXYS rats were randomly distributed in two groups (n = 15) and were injected intramuscularly (femoral quadriceps) with p62DNA in dose 150 mkg per rat in 60 µl (Elenagen, 2.5 mg/ml) on PBS or with only PBS (n = 15). All groups were subjected to nine injections at one-week intervals. The animals received the last injection at the age of 4 months. An ophthalmologist examined all animals five times: before treatment at the age 1.5 months and at the ages 4, 6, 8, and 10,5 months, respectively.

### Ophthalmoscopic examination

All rats underwent funduscopy with a Heine BETA 200 TL Direct Ophthalmoscope (Heine, Herrsching, Germany) after dilatation with 1% tropicamide. An assessment of stages of retinopathy was performed according to the Age-Related Eye Disease Study grade protocol (AREDS; http://eyephoto.ophth.wisc.edu). The degree of retinopathy was estimated as follows: 0 arbitrary units (a.u.) corresponds to healthy retina; 1 a.u., appearance of drusen and other pathological changes in the RPE and partial atrophy of the choroid capillary layer; 2 a.u., exudative detachment of RPE and of retinal neuroepithelium, with further choroid capillary layer atrophy; and 3 a.u., neovascularization and exudative-hemorrhagic detachment of RPE and neuroepithelium scarring.

### Antibodies

Mouse monoclonal anti-p62 (ab56416), rabbit polyclonal anti-Gfap (ab7260), rabbit polyclonal anti-actin (ab1801), goat polyclonal anti-Iba1 (ab5076), mouse monoclonal anti-Cd68 (ab31630), and a secondary antibody – a donkey anti-goat IgG H&L antibody (conjugated with Alexa Fluor® 488; ab150129), donkey anti-rabbit IgG H&L antibody (conjugated with Alexa Fluor® 488; ab150073), donkey anti-mouse IgG H&L antibody (conjugated with Alexa Fluor® 568; ab175472) and goat anti-rabbit IgG H&L antibody (HRP; ab6721) were acquired from Abcam (Cambridge, UK).

### RPE flat-mount staining

As described by previous work [12,17], the enucleated eyes with an incision along the limbus were fixed in 4% paraformaldehyde in phosphate-buffered saline (PBS) for 2 h. The anterior segment of the eye (cornea, iris, ciliary body and lens) was removed. Retinal tissue was carefully excised from the eyecup, and the remaining cups containing RPE, choroid, and sclera were thoroughly washed in PBS with 0,1% Triton X-100 (PBST) and dissected into quarters by radial cuts. The RPE/choroid flat mounts were incubated in PBS/bovine serum albumin (BSA) 5% with 1% Triton X-100 for 1 h for blocking and permebilization. Next, the samples were stained with fluorescein isothiocyanate (FITC)-phalloidin (1:100, P5282, Sigma-Aldrich) at 4°C 48 h to visualise the cytoskeleton and cell shapes during en face imaging. After washes in PBST, the RPE/choroids were flat-mounted on glass slides and were coverslipped with the Fluoro-shield mounting medium containing 4′,6-diamidino-2-phenylindole (DAPI; ab104139, Abcam). Images were acquired with a confocal microscope (LSM 780 NLO, Zeiss).

### Western blotting

Immunoblotting was performed as previously described [8]. To measure P62 protein level we used retinas obtained from six rats for group. The retina was separated from the other tissues, placed in microcentrifuge tubes for protein isolation, and frozen in liquid nitrogen. The retinas from the left and right eyes of one rat were mixed. Frozen tissues of retina were homogenized in protein lysis buffer radioimmunoprecipitation assay (50 mmol/L Tris-HCl, pH 7.4; 150 mmol/L NaCl; 1% Triton X-100; 1% sodium deoxycholate; 0.1% SDS; and 1 mmol/L EDTA) supplemented with protease inhibitor cocktail (P8340; Sigma-Aldrich, St. Louis, MO). After incubation for 20 minutes on ice, samples were centrifuged at 9660 × g at 4°C for 30 minutes and the supernatants were transferred to new tubes. Total proteins were measured with a Bio-Rad Bradford kit (Bio-Rad Laboratories). Samples were resolved on 12% SDS-PAGE on Tris-glycine buffer (25 mmol/L Tris base, 190 mmol/L glycine, and 0.1% SDS) and transferred to nitrocellulose membranes. Antibodies and dilutions used in immunoblotting included an anti-P62 antibody (1:1,000) and anti-β-actin antibody (1:1,000). After blockage with 5% bovine serum albumin (BSA; Sigma-Aldrich) in 0.01 M phosphate buffer with 0.1% Tween-20 (PBS-T) for 1 h, the membranes were incubated with the primary antibodies at 4°C overnight. After incubation with the respective secondary antibody (1:3,000), chemiluminescent signals were measured and scanned, and intensity of the bands was quantified in the ImageJ software (NIH, Bethesda, MD, USA). β-Actin served as an internal loading control.

### Immunohistochemistry

Immunofluorescent staining was performed according to the standard method as described [5]. The eyes were removed and fixed in fresh 4% paraformaldehyde in PBS for 2 h, washed three times in PBS, and then cryoprotected in graded sucrose solutions (10%, 20%, and 30%). Posterior eyecups were embedded in Tissue-Tek® O.C.T. Compound (Sakura), frozen, and stored at −80°C. Tissue slices (10 µm thick) were prepared on a Microm HM-505N cryostat at –20°C, transferred onto Polysine® glass slides (Menzel-Glaser), and stored at −20°C. Primary antibodies and dilutions were as follows: anti-Iba1 (1:250), anti-Gfap (1:250), and anti-Cd68 (1:300). Primary antibodies were incubated for 18 h at 4°C. After incubation with the respective secondary antibodies diluted 1:300 for 1 h at room temperature, the tissue slices were coverslipped with the mounting medium containing DAPI (ab104139, Abcam) and were examined under the Zeiss microscope Axioplan 2. The negative control samples with the omitted primary antibody emitted only a minimal autofluorescent signal. For each image acquisition, all imaging parameters were the same. The morphometric parameters (the retinal thickness, Iba1^+^ and Cd68^+^ cell quantifications) were measured using quantitative analyses of the images performed with Axiovision software (SE64 4.9.1). Estimation was performed by examination of the five fields of view for each retina.

### Statistical analysis

The data were analyzed using repeated-measures ANOVA (analysis of variance) and nonparametric tests using the statistical package (Statistica 8.0 software). One-way analysis of variance was used for individual group comparisons. The Newman–Keuls test was applied to significant main effects and interactions in order to assess the differences between some sets of means. To assess the therapeutic effectiveness, we performed a dependent pairwise comparison of the eye states before and after treatment (t-test for dependent samples). The data of Iba+ and Cd68+ cells counting were analyzed by nonparametric method (the Mann–Whitney U test). The data are presented as mean ± SEM. The differences were considered statistically significant at p<0.05.
